# Effect of Salinity and Silicon Doses on Onion Post-Harvest Quality and Shelf Life

**DOI:** 10.3390/plants11202788

**Published:** 2022-10-20

**Authors:** Jefferson Bittencourt Venâncio, Nildo da Silva Dias, José Francismar de Medeiros, Patrícia Lígia Dantas de Morais, Clístenes Williams Araújo do Nascimento, Osvaldo Nogueira de Sousa Neto, Luciara Maria de Andrade, Kleane Targino Oliveira Pereira, Tayd Dayvison Custódio Peixoto, Josinaldo Lopes Araújo Rocha, Miguel Ferreira Neto, Francisco Vanies da Silva Sá

**Affiliations:** 1 Center for Agrarian Sciences, Federal Rural University of the Semi-Arid Region, Mossoró 59625-900, Brazil; 2 Department of Agronomy, Federal Rural University of Pernambuco, Recife 52171-900, Brazil; 3 Department of Engineering, Federal Rural University of the Semi-Arid Region, Angicos 59515-000, Brazil; 4 Center for Agro-Food Science and Technology, Federal University of Campina Grande, Pombal 58840-000, Brazil

**Keywords:** *Allium cepa* L., mineral nutrition, horticulture, soil fertility, diatomaceous earth, *Melosira granulate*

## Abstract

Salt stress during pre-harvest limits the shelf life and post-harvest quality of produce; however, silicon nutrition can mitigate salt stress in plants. Thus, we evaluated the effects of salinity and fertilization with Si, in pre-harvest, on the morpho-physiological characteristics of onion bulbs during shelf life. The experiment was set up in randomized complete blocks, with treatments arranged in split-split plots. The plots had four levels of electrical conductivity of irrigation water (0.65, 1.7, 2.8, and 4.1 dS m^−1^). The subplots had five fertilization levels with Si (0, 41.6, 83.2, 124.8, and 166.4 kg ha^−1^). The sub-sub plots had four shelf times (0, 20, 40, and 60 days after harvest). Irrigation water salinity and shelf time reduced firmness and increased the mass loss of onion bulbs during shelf life. Salt stress reduced the contents of sugars and total soluble solids of onion bulbs during storage; however, Si supply improved the contents of these variables. Salinity, Si supply, and shelf time increased the concentrations of pyruvic and ascorbic acids in onion bulbs during shelf life. Si doses between 121.8 and 127.0 kg ha^−1^ attenuated the impacts caused by moderate salinity, increasing the synthesis of metabolites and prolonging the onion bulbs’ shelf life.

## 1. Introduction

Onion (*Allium cepa* L.) is a vegetable appreciated worldwide for its food, nutritional and medicinal characteristics [1,2]. Onions are in high demand all year, so the bulbs are usually stored due to logistical limitations and the seasonality of crop harvests [3].

Pre-harvest aspects of crop management techniques, such as mineral nutrition, irrigation, cultivar, or other agronomic conditions, affect the post-harvest conservation parameters, processing, and quality of onion bulbs. Post-harvest factors contributing to storage performance include the method and duration of curing, grading, packaging method, and storage environment [4]. The significant post-harvest losses of onions are mainly caused due to bulb sprouting and rotting, which contribute to loss in storage life and quality [5]. The market value is predominantly related to bulb firmness and dry matter content [6]. To supply customers and processors with high-quality, firm onions with a high dry matter content devoid of sprouts, secondary roots, and illnesses, mature bulbs are cured, dried, and stored in cool rooms between seasons [7]. Sugars and organic acids contribute to the organoleptic test and distinct flavor and aroma [8]. Biotic and abiotic stresses affect post-harvest conservation, such as pathogen incidence, exposure to temperature extremes, solar radiation, light, winds, and salinity [9,10].

Salt stress through acclimatization programs regulate plant development, physiology, and metabolism [11]. Redox metabolism and cellular osmoregulation are altered at the gene expression level to produce antioxidant and compatible molecules. Studies have examined that plants under salt stress increase their sugars, proline, betaine, glycine, polyamines, ascorbate, glutathione, tocopherols, carotenoids, thiols, and flavonoid levels in tissues; on the other hand, they decrease fresh bulb weight, production of large bulbs, bulb yield, the quality of onion bulbs, bulb firmness and bulb pH [12,13,14,15,16,17]. The literature reveals that salt stress alters production levels of proline, phenolic compounds, and pyruvic acid precursors in onions [17,18,19].

There is a need to evaluate salt stress effects on metabolite production related to onion bulb quality and conservation, such as pH, ascorbic acid, sugars, soluble solids, and titratable acidity.

Silicon is an element considered beneficial to plants [20]. Si improves plant acclimatization to multiple stressors, such as salt stress [21]. In salt stress, Si fertilization improves the essential plant nutrient uptake, antioxidant defense systems, and solute and plant hormone production [20]. In onions, ref. [22] showed that fertilization with Si, in interactions with zeolite and selenium (Se), improved some bulbs’ qualitative characteristics, such as the large-bulb production, bulb-dry matter, soluble solids, and protein content. Refs. [17,22] observed that Si improved the physiological (enzymatic activity, chlorophyll levels, and photosynthetic activity) and nutritional (increase in nitrogen, nitrate, and potassium concentrations; decrease in sodium concentration) of the quality of onion bulbs, and plant salt tolerance under conditions of saline soil and brackish irrigation water. However, Si fertilization effects on shelf life and post-harvest characteristics of onion bulbs grown under saline stress are less well known.

We hypothesized that Si fertilization improves the post-harvest quality and shelf life of onion bulbs grown under saline stress. In this study, we examined the effects of Si fertilization on the post-harvest quality and shelf life of onion bulbs grown under increasing salinity levels of irrigation water.

## 2. Results

### 2.1. Bulb Firmness and Mass Loss

The general analysis of variance (ANOVA) showed an interaction between the salinity of irrigation water (EC) and shelf life (SL), and a non-significant effect of the Si dose (SD) on the variables bulb firmness (BF) and mass loss (ML).

**Figure 1:**
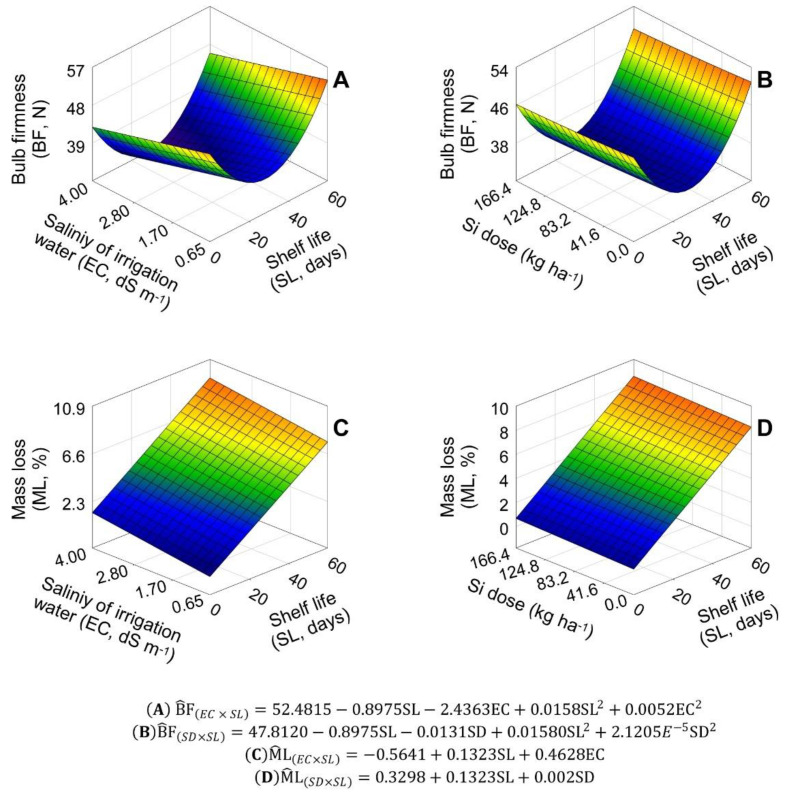
Unfolding of the treatment factors of salinity of irrigation water (EC) and silicon doses in the soil (SD), with shelf life (SL), for the variables: bulb firmness (FB, in N) (**A**,**B**); and mass loss (ML, %) (**C**,**D**), in ‘Rio da Antas’ onion bulbs.

The BF of ‘Rio das Antas’ onions was significantly reduced with increases in EC and SL, being described by the nonlinear parabolic model (Figure 1A). The minimum BF of 30.07 N was reached at 28.4 shelf days under an irrigation water salinity of 4.0 dS m^−1^. The ML of onion bulbs, on the other hand, increased significantly with increases in EC and SL, being described by the nonlinear flat model (Figure 1C). The maximum ML of 9.23% was reached at 60 shelf days, with an EC of 4.0 dS m^−1^.

Fertilization with Si did not significantly influence onion BF and ML. However, the significant effects of the SL factor on these variables allowed fits of three-dimensional parabolic and flat models, respectively, to the SD × SL interaction analysis data (Figure 1B,D). Minimum BF values of 33.47 and 35.07 N were reached at 28.4 shelf days with Si doses ranging from 0 to 166.4 kg ha^−1^ (Figure 1B). Maximum ML values of 8.27 and 8.60% were reached at 60 shelf days with the same Si doses (Figure 1D).

### 2.2. Tunic Color

Irrigation water salinity and Si fertilization failed to the color of onion bulbs. However, shelf time affected bulb color, altering the CIE color parameters L*, a*, b*, C*, and °H*.

**Figure 2:**
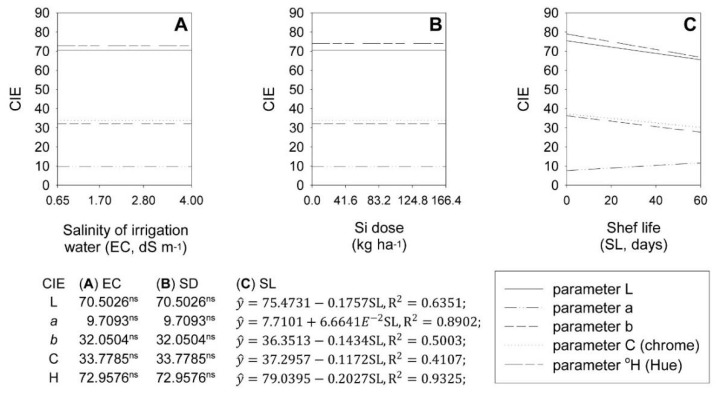
Effect of increasing salinity (EC) (**A**), silicon doses (SD) (**B**), and shelf life (SL) (**C**) on the coloration of ‘Rio das Antas’ onion bulbs, parameterized according to CIE standard L*, a*, b*, C*, and °H.

For the EC and SD factors, the CIE parameters L*, a*, b*, C*, and °H were 70.50, 9.71, 32.05, 33.78, and 72.96, respectively (Figure 2A,B). For SL, for each storage day, there was a linear reduction of 0.2328, 0.3945, 0.3142, and 0.2565% in the CIE parameters L*, b*, C*, and H*, respectively, in addition to a linear increase of 0.8643% in the CIE parameter a* (Figure 2C).

### 2.3. Soluble Sugars and Total Soluble Solids

**Figure 3:**
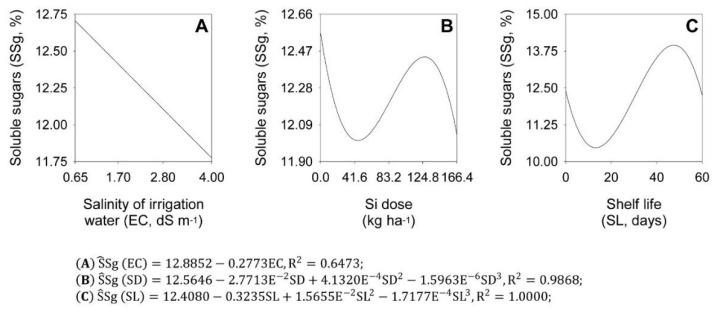
Effect of salinity levels (EC) (**A**), silicon doses (SD) (**B**), and shelf life (SL) (**C**) on total soluble sugars (SSg) in ‘Rio das Antas’ onion bulbs.

There was a significant main effect of the treatment factors EC, SD, and SL on soluble sugars (SSg). Regression analyses showed that EC, SD, and SL differently affected the metabolism of soluble sugars during shelf life, with fits of linear, cubic, and cubic models, respectively (Figure 3).

Salinity decreases onion bulb SSg content during shelf life. Starting from 12.89% SSg, the increase in salinity caused a decrease at a rate of 2.15% for each EC unit added to the irrigation water (Figure 3A). In Si fertilization, the minimum SSg content was 12.01%, obtained with a Si dose of 45.6 kg ha^−1^, while the maximum was 12.44%, obtained with a Si dose of 127.0 kg ha^−1^ (Figure 3B). In shelf life, the minimum SSg content was 10.47% at 13.2 shelf-life days, while the maximum was 13.95% at 47.6 days (Figure 3C).

**Figure 4:**
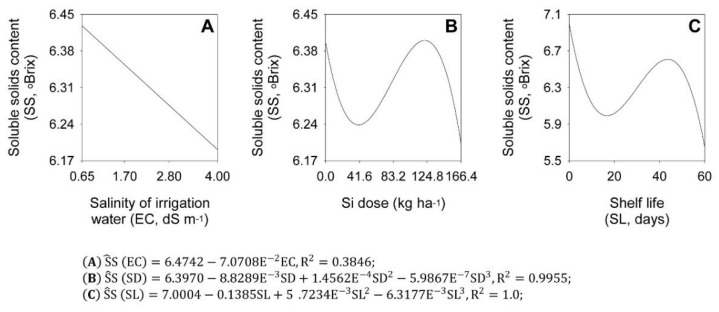
Effect of salinity levels (EC) (**A**), silicon doses (Si) (**B**), and shelf life (SL) (**C**) on soluble solids content (SS) variable in ‘Rio das Antas’ onion bulbs.

Figure 4 shows the main effect of the EC, SD, and SL on the total soluble solids (SS). The salinity of irrigation water decreased onion bulb SS content during shelf life. Based on the initial SS content of 6.47 °Brix, there was a decrease of 1.09% for each EC unit added to the irrigation water (Figure 4A). However, Si fertilization caused cubic variation in the onion bulb SS content during shelf life, being: 6.40 °Brix, in the control treatment; 6.24 °Brix, with the Si dose of 40.4 kg ha^−1^; 6.40 °Brix, with the Si dose of 121.8 kg ha^−1^; and 6.20 °Brix, with the Si dose of 166.4 kg ha^−1^ (Figure 4B). For shelf life, the increase in the exposure time of the bulbs caused cubic variation in SS, being: 7.00 °Brix, at the beginning of shelf life; 5.99 °Brix, at 16.7 shelf days; 6.62 °Brix, at 43.7 shelf days; and 5.65 °Brix, at 60 shelf days (Figure 4C).

### 2.4. pH, Titratable Acidity, and SS/TA Ratio in Bulbs

**Figure 5:**
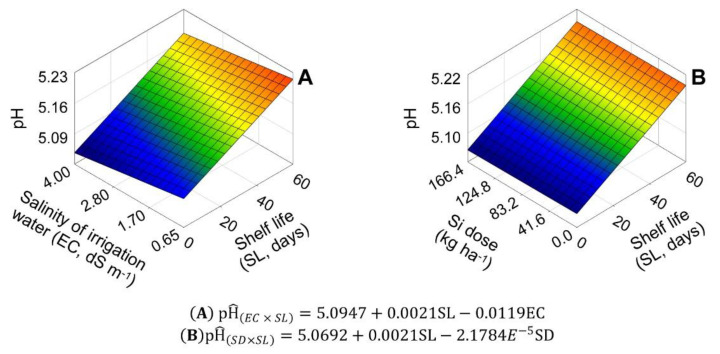
Unfolding of the treatment factors of salinity of irrigation water (EC) (**A**) and silicon dose (SD) (**B**), with shelf life (SL), for the hydrogenic potential (pH) variable in ‘Rio da Antas’ onion bulbs.

Figure 5 shows pH responses to EC × SL and SD × SL interactions. To EC × SL interaction, the basal pH was 5.09. EC increase caused a pH decrease of 0.2336% for each EC unit. SL increase caused a pH increase of 0.0412% for each shelf-life day (Figure 5A). For SD × SL interaction, the basal pH was 5.07. SD did not change pH significantly, but the SL increase caused an increase of 0.04114% for each shelf-life day (Figure 5B).

**Figure 6:**
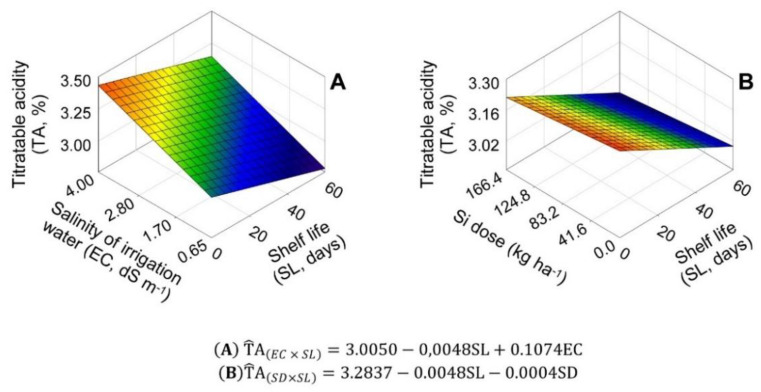
Unfolding of the treatment factors of salinity of irrigation water (EC) (**A**) and silicon dose (Si) (**B**), with shelf life (SL), for the variable titratable acidity (TA, %), in ‘Rio da Antas’ onion bulbs.

**Figure 7:**
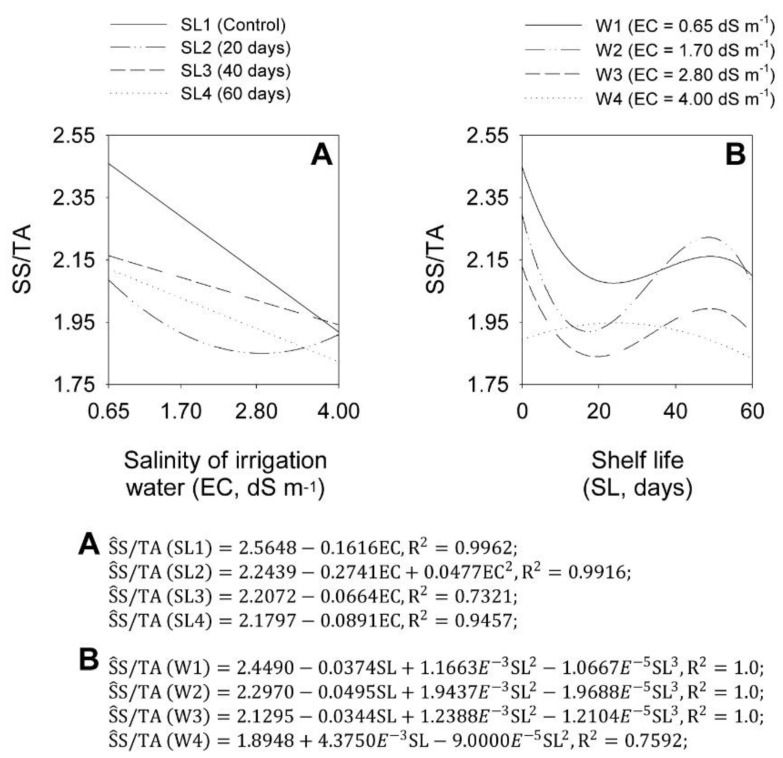
Effect of the interaction between salinity of irrigation water levels (EC) (**A**) and shelf life (SL) (**B**) on the variable SS/AT in ‘Rio da Antas’ onion bulbs.

**Figure 8:**
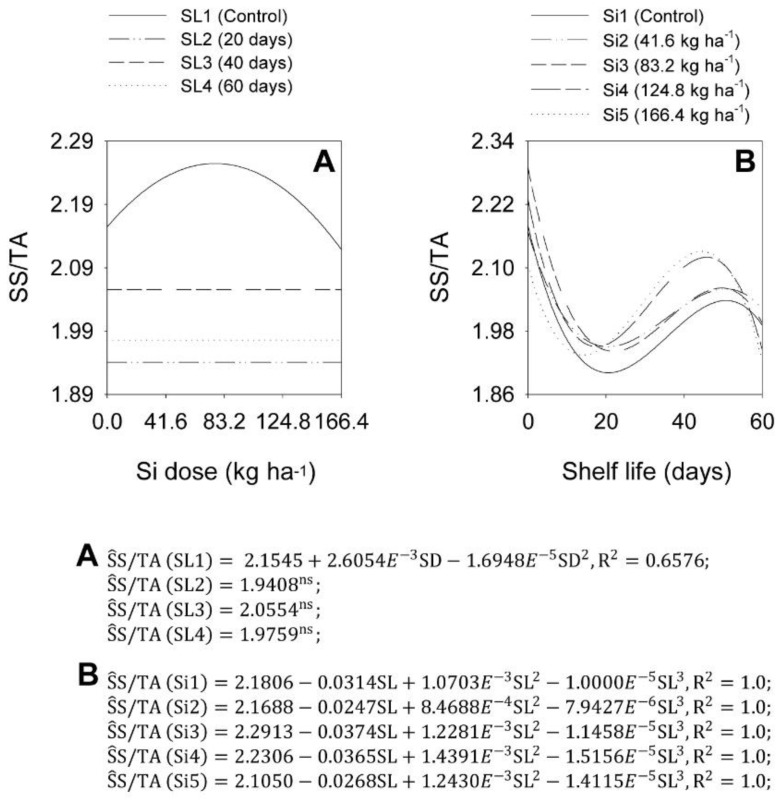
Effect of the interaction between Si dose levels (SD) (**A**) and shelf life (SL) (**B**) on the variable SS/AT in ‘Rio da Antas’ onion bulbs.

The variable TA and SS/TA ratio showed significant effects for the parameters related to EC and SL, but there was no parameterization for SD (Figure 6, Figure 7 and Figure 8). For EC × SL interaction, basal TA was 3.01%. The salinity increase caused a TA increase of 3.57% for each EC unit increased, and the SL increase caused a TA decrease of 0.16% for each day of shelf life (Figure 6A). For SD × SL interaction, the initial TA was 3.28%. SD did not change TA significantly, but the SL increase caused a TA decrease of 0.15% for each shelf-life day (Figure 6B).

EC analyses at each SL showed that the salinity increase decreased the onion bulb SS/TA ratio, depending on shelf life. At SL1, starting from the initial SS/TA ratio of 2.56, each EC unit increase caused a linear decrease of 6.30%. At SL2, the minimum SS/TA ratio value of 1.85 occurred in the EC of 2.87 dS m^−1^. At SL3 and SL4, each EC unit increase caused a linear decrease of 3.00 and 4.09%, starting from the initial SS/TA ratio of 2.21 and 2.18, respectively (Figure 7A).

SL analyses at each EC showed that the SL increase affected the onion bulb SS/TA ratio (Figure 7B). At salinity levels W1, W2, and W3, the SS/TA ratio was at its maximum at the beginning of the shelf period, being 2.45, 2.30, and 2.13, decreasing to 2.08, 1.92, and 1.84, at 23.8, 17.3 and 19.4 shelf life days, increasing to 2.16, 2.22 and 1.99 at 49.1, 48.6 and 48.8 days, and decreasing to minimum values of 2.1, 2.07 and 1.91 at 60 days, respectively. At W4, the initial SS/TA ratio was 1.89, reaching a maximum of 1.95 at 24.3 shelf-life days and decreasing to 1.83 at 60 days.

SD analyses at each SL showed that the SD increase effects on the onion bulb SS/TA ratio were significant at SL1 and not significant at SL2, SL3, and SL4. At SL1, the maximum SS/TA ratio value of 2.25 occurred at a Si dose of 76.9 kg ha^−1^. At SL2, SL3, and SL4 levels, average SS/TA ratio values were 1.94, 2.06, and 1.98, respectively (Figure 8A). SL analyses at each SD showed that the SL increase decreased the onion bulb SS/TA ratio, depending on the Si dose (Figure 8B). At Si1, Si2, Si3, Si4, and Si5 levels, the SS/TA ratios were at a maximum at the beginning of the shelf life, being 2.18, 2.17, 2.29, 2.23, and 2.11, decreasing to 1.90, 1.95, 1.94, 1.95, and 1.93 at 20.6, 20.4, 21.5, 17.6, and 14.2 shelf life days, and increasing to 2.04, 2.06, 2.06, 2.12, and 2.13 at 50.7, 50.6, 49.5, 45.7, and 44.5 days, decreasing to minimum SS/TA ratio values of 1.99, 2.02, 2.00, 195, and 1.92 at 60 days, respectively.

### 2.5. Concentrations of Pyruvic and Ascorbic Acids

**Figure 9:**
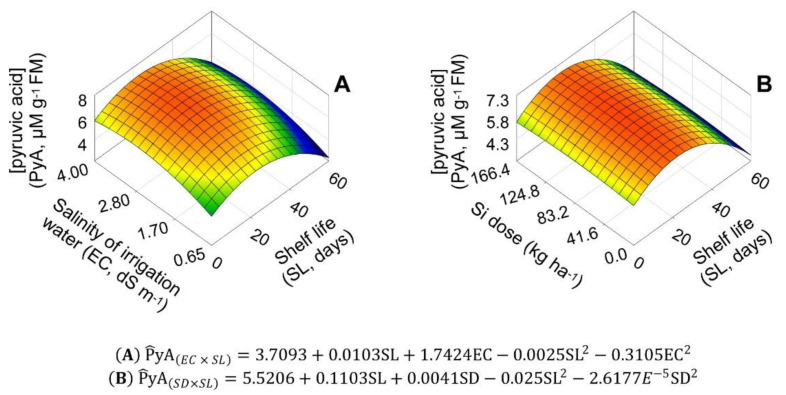
Unfolding of the treatment factors of salinity of irrigation water (EC) (**A**) and silicon dose (SD) (**B**), with shelf life (SL), for the variable pyruvic acid concentration (PyA, µM g^−1^ FM) in ‘Rio da Antas’ onion bulbs.

The PyA responses to EC × SL and DS × SL interaction were described by a three-dimensional parabolic model, with maximum PyA concentrations of 6.93 and 6.75 μM g^−1^ of FM at EC and SD of 2.81 dS m^−1^ and 78.3 kg ha^−1^, respectively, both at 22.1 shelf life days (Figure 9A,B).

**Figure 10:**
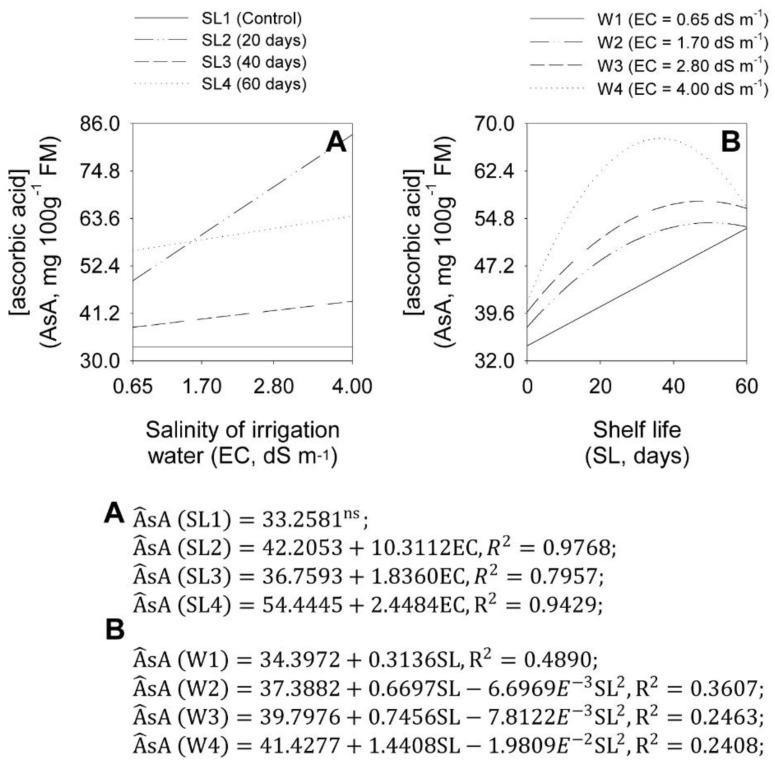
Effect of the interaction between salinity of irrigation water levels (EC) (**A**) and shelf life (SL) (**B**) on ascorbic acid (AsA) concentration in ‘Rio da Antas’ onion bulbs.

EC analyses at each SL showed that the EC increases increased onion bulb AsA concentration, depending on the onion shelf life (Figure 10A). At SL1, there was no significant effect of the increase in EC on onion bulb AsA concentration, which was 33.23 mg 100 g^−1^ of FM. At SL2, starting from the initial AsA of 42.21 mg 100 g^−1^ of FM, each EC unit increase caused a linear increase of 24.43% in the onion bulb AsA concentration. At SL3, starting from the initial AsA of 36.76 mg 100 g^−1^ of FM, each EC unit increase caused a linear increase of 4.99% in the onion bulb AsA concentration. At SL4, starting from the initial AsA of 54.44 mg 100 g^−1^ of FM, each EC unit increase caused a linear increase of 4.50% in the onion bulb AsA concentration.

SL analysis at each EC showed that the SL increases increased the onion bulb AsA concentration, depending on the salinity level (Figure 10B). At W1, starting from the initial AsA of 34.40 mg 100 g^−1^ of FM, the SL increase caused a linear increase of 0.91% in the concentration. At W2, the increase in EC caused a quadratic increase in AsA, which reached a maximum of 54.13 mg 100 g^−1^ of FM at 50 shelf-life days. At W3, the increase in EC caused a quadratic increase in AsA, with a maximum value of 57.59 mg 100 g^−1^ of FM at 47.7 shelf life days. At W4, the EC increase caused a quadratic increase in AsA, which reached a maximum AsA value of 67.63 mg 100 g^−1^ of FM at 36.4 shelf-life days.

**Figure 11:**
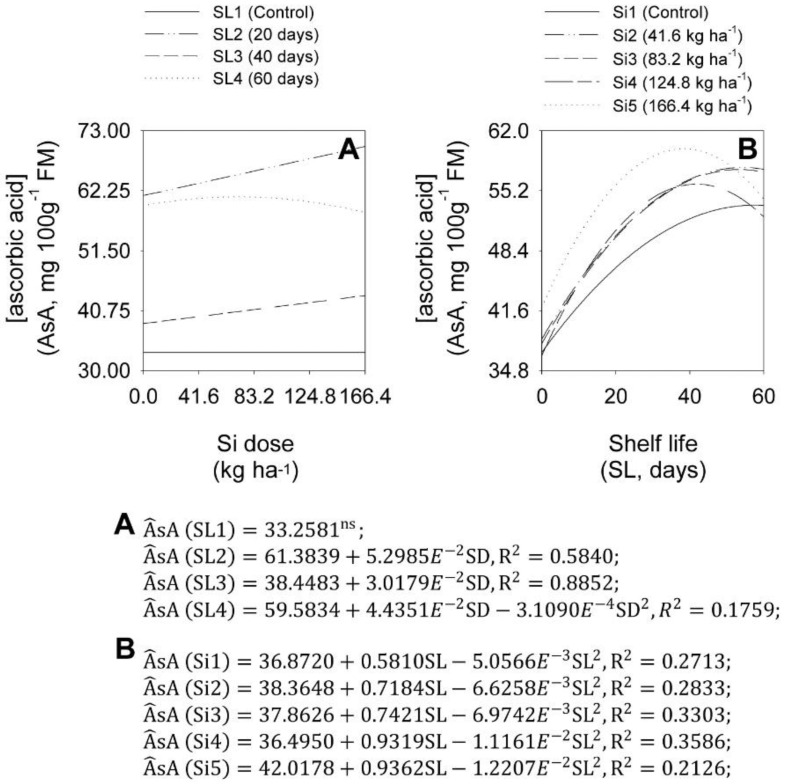
Effect of the interaction between Si dose levels (SD) (**A**) and shelf life (SL) (**B**) on ascorbic acid concentration (AsA) in ‘Rio da Antas’ onion bulbs.

SD analyses at each SL showed that the SD increases increased onion bulb AsA concentration, depending on the shelf life (Figure 11A). At SL1, there was no significant effect of SD increase on onion bulb AsA concentration, on average, 33.23 mg 100 g^−1^ of FM. At SL2, starting from the initial AsA of 61.38 mg 100 g^−1^ of FM, each SD increase caused a linear increase of 0.0863% in the onion bulb AsA concentration. At SL3, starting from the initial AsA of 38.45 mg 100 g^−1^ of FM, each SD increase caused a linear increase of 0.0785% in the onion bulb AsA concentration. At SL4, the SD increase increased onion bulb AsA concentration, up to a maximum AsA of 61.17 mg 100 g^−1^ of FM, with 71.3 kg ha^−1^.

SL analysis at each SD showed that the SL increase quadratically increased the onion bulb AsA concentration, depending on the Si dose (Figure 11B). At Si1, the SL increase caused a quadratic increase in AsA, which reached a maximum AsA value of 53.56 mg 100 g^−1^ of FM at 57.5 shelf-life days. At Si2, the SL increase caused a quadratic increase in AsA, with a maximum AsA of 57.84 mg 100 g^−1^ of FM at 54.2 shelf-life days. At Si3, the SL increase caused a quadratic increase in AsA, with a maximum AsA of 57.60 mg 100 g^−1^ of FM, at 53.2 shelf-life days. At Si4, the SL increase caused a quadratic increase in AsA, with a maximum AsA of 55.95 mg 100 g^−1^ of FM at 41.8 shelf-life days. At Si5, the SL increase caused a quadratic increase in AsA, with a maximum AsA of 59.97 mg 100 g^−1^ of FM at 38.3 shelf-life days.

## 3. Discussion

Salt stress affects different parameters in onions. The literature shows that irrigated onion yield with 0.65 dS m^−1^ water is 99.6 Mg ha^−1^ and decreases to 64.4 Mg ha^−1^ when irrigated with 4.00 dS m^−1^, representing a yield loss of 35.3% [17]. The authors showed that Si does not increase irrigated onion yield with 4.00 dS m^−1^ water but improves the irrigated onion yield with 1.7–2.8 dS m^−1^ water, mainly producing of Class 3 bulbs (50 ≤ Φ < 70 mm). Onions irrigated with 1.7 dS m^−1^ and fertilized with 166.4 kg ha^−1^ of Si produce 93.8 t ha^−1^. However, onions irrigated with water of 2.8 dS m^−1^ only responded up to 78.5 kg ha^−1^ of Si with a production of 81.2 t ha^−1^ [17]. The onions fertilized with Si and irrigated with water of 1.7–2.8 dS m^−1^ decreased the yield by 5.8–18.5% [17]. The salinity of 1.7–4.0 dS m^−1^ reduced onion yield, but few studies verify the effect of salinity on onion shelf life. There is a need to evaluate salt stress effects on the metabolite production related to onion bulb quality and conservation, such as pH, ascorbic acid, sugars, soluble solids, and titratable acidity. We examined the effects of Si fertilization on the post-harvest quality and shelf life of onion bulbs grown under increasing salinity levels of irrigation water.

Our results showed that irrigation with saline water decreased the firmness of onion bulbs. Onion bulbs have high firmness at harvest because of the endogenous uronic acid concentrations and the relationship between total and water-soluble pectins [23]. However, salt stress caused oxidative damage to the aerial part of the onion and decreased the relative water content and membrane stability index in leaf and bulb tissues [1,24]. These changes could decrease the firmness levels of onion bulbs. Membrane stability loss promotes electrolyte leakage and oxidative and hydrolyzing reactions by contact between enzymes and substrates [9]. Low relative water content in bulb tissues causes pressure and intercellular cohesion loss, detaching the middle lamella from the cell walls by shear forces [25].

In storage, an increase in onion BF occurred after the 28th shelf day due to the elastic properties of the epidermal tissues of the bulb cataphylls [26], which may increase the resistance to penetration in onion bulbs due to the decrease in turgor cells resulting from the loss of moisture from the bulbs during their shelf life.

Mass loss (ML) in onions occurs due to transpiration water loss from the bulb tissues [27,28]. We observed that an increase in irrigation water salinity significantly increased the ML of ‘Rio das Antas’ onions, compromising its shelf life. Salinity-induced osmotic stress in the pre-harvest caused a water deficiency in onion bulbs after harvest, leading to an increase in the rate of cell membrane dehydration during shelf life [29]. In general, salinity increases the production of ROS in intra- and intercellular spaces, resulting in cell death, electrolyte leakage, senescence, and increased dehydration of plant tissues in fresh food products [29,30]. Although salinity increased the ML of onion bulbs during conservation, the maximum ML of 9.23%, reached with EC of 4.00 dS m^−1^ at the end of 60 days of storage, was slightly lower than the maximum tolerable mass loss of 10% before the onion is considered non-marketable [29]. Water loss reduces the shelf life of vegetables, revealing changes in qualitative characteristics related to texture, such as softening and visible wilting, which are essential in indicating the deterioration of fresh products [6,29,31].

Onion bulbs can be stored for long periods (up to 8 months) when kept under refrigeration conditions (temperature, 2 °C) and high relative humidity (RH, 98%) [7,28]. In this experiment, we verified that each conservation day increased by 0.1323% in ML under an average air temperature of 29.5 ± 0.7 °C and RH of 67 ± 5%. Since transpiration is directly proportional to the water vapor gradient between the surface of the plant and the surrounding air [29], storage environment conditions influenced the ML.

Si-accumulating plants deposit Si in the root endoderm, leaf epidermis, and leaf cuticle in the form of hydrated amorphous silica (SiO_2_.nH_2_O), close to the cell walls to form alternative polyphenolic complexes to lignin [32,33,34]. Thus, the remarkable properties of Si in plants confer its potential capacity to increase the resistance of their cell walls [20] and reduce the transpiration processes of the tissues by improving interfaces of resistance to transpiration close to the cuticular layers [31]. In this study, the Si increase in soil fertilization did not significantly alter the resistance (BF) and ML of onion bulbs. The beneficial effects of Si on shoot tissues may not occur in onion bulbs because shoots of this species fail to accumulate Si even at high levels in the soil [32]. Ref. [22] observed that the increase in Si fertilization did not significantly alter the concentrations of Si in the aerial part of onion plants and, according to [33], the processes of silicification of the cell walls in the aerial part of the plants depend on the species and are specific to each type of cellular tissue. So far, we only know that onions respond to fertilization with Si, increasing its content and improving the anatomy of the roots [32].

The L*, b*, and C* reductions indicate a darkening yellow color tone decrease and saturation loss of five onion bulbs along shelf life. On the other hand, the increase in a* and hue angle (°H) reduction indicates that the shelf time increased the red color tone of the bulbs. The b* and C* reductions in the bulbs suggest degradation of quercetin phenolic compounds during onion shelf life. Ref. [35] reported that increased concentrations of flavonoids such as quercetin 7,4-diglucoside, quercetin 3,4-diglucoside, quercetin 3-glucoside and quercetin 4-glucoside are responsible for conferring yellow color to the onion tunic. Quercetin compartmentalization in the transition zones between the living and dead cells of the epidermis suberization process and flavonol aglycone degradation occurs by a self-catalytic oxidation process triggered by the generation of radical molecules of quercetin and superoxide radical (O_2_^•−^) [36].

The °H angle reduction and a* increase occur due to the onion tunic darkening observed during the bulb self-life. We found that the cured yellow onions show color changes in the tunic of the bulbs, from light whitish brown to dark reddish-brown, concomitantly with the gradual reduction in the hue angle (°H) [28]. Nonetheless, [37] found that the hue angle of the tunic is negatively correlated with the contents of anthocyanin and total flavonoids in red onions (‘Red Baron’). However, this correlation fails to be observed in the yellow onion cultivars (‘Sherpa’ and ‘Wellington’) [37].

Salinity increases mitochondrial ROS production and the generation of free radical molecules in harvested products [30]. In contrast, the soluble sugar degradation can feed the NADPH-producing metabolic pathways, as in the oxidative way of phosphate pentoses, contributing to the elimination of ROS and regulation of cellular redox [38,39] in several pathways of the antioxidant defense system, such as the recovery of enzymatic cofactors of the Asada-Halliwell cycle and the feeding of the proline biosynthesis pathway [40,41,42]. Thus, higher soluble sugar consumption and production in plants grown under high salinity conditions occur due to their multifaceted function in the physiological responses of plants to abiotic stress [43]. After harvest, however, sink organs are disconnected from their respective sources of sugars and begin the processes of respiratory degradation of carbohydrates and senescence [44,45]. However, in shelf life, products grown under pre-harvest salinity conditions, in addition to exhibiting the natural catabolism of sugars in senescence and respiration processes, exhibit exacerbated energy consumption due to the regulation of osmoprotective metabolism, antioxidant (ROS eliminator) metabolism, and balance of cellular redox [12,30,39].

Carbohydrate metabolism responses to the nutritional Si supply vary widely with the species, genetic material within the species, specific Si nutritional habits, and environmental conditions [46,47,48,49,50]. Refs. [46,47] found that Si increased the concentration of soluble sugars in plants under salinity conditions, reducing cellular stresses caused by osmotic and ionic stresses. This effect occurs due to the lower catabolic rate of soluble sugars in plants treated with Si than in untreated plants. Although our results showed oscillation in SSg content as a function of Si doses.

The reduction of SSg in the first shelf-life days is possibly due to respiratory consumption and ethylene production, which may have been initially accelerated in response to stimuli related to the regeneration of lesions that occurred in the harvesting process [44,51]. In addition, the conditions of the atmosphere of the storage system (average air temperature of 29.5 ± 0.7 °C and relative humidity of 67 ± 5%) were favorable to trigger enzymatic activities and, consequently, cellular energy consumption [44], which possibly contributed to the higher initial consumption of SSg during the onion’s shelf life.

The increase in the SSg content of bulbs between 13.2 and 47.6 shelf-life days may be related to increased activity of cellulase and enzymes that solubilize peptic substances, as well as other enzymes that degrade carbohydrates, converging to cellulose degradation and conversion into glucose [44]. Ref. [23] found a 40% reduction in cellulose concentrations and changes in the activities of wall-modifying enzymes (polygalacturonase and pectin methyl esterase) in onion strains under storage conditions, which was related to decreased cell wall resistance. In addition, there was a significant increase in soluble solids content concomitantly with the reduction of cellulose content in onion strain M87-WOPL, indicating probable degradation of cellulose into sugars in stored bulbs.

SS is the solid cellular compound found in a plant juice aliquot, detectable by diffractometry calibrated using a sucrose solution. Although there is a significant correlation between the obtained values and the sucrose solution, solids include several organic and inorganic components, such as carbohydrates, organic acids, proteins, fats, and minerals. Ref. [52] found that the increase in salinity in the cultivation solution did not cause significant changes in the SS content of onion bulbs after harvest. On the other hand, Ref. [53] found that the SS content of the bulbs increased quadratically as a function of decreasing periods of salt stress duration during the phenological cycle of the plant. Concerning fertilization with Si, Ref. [22] reported an increase in the SS content of bulbs after harvesting due to increased doses of Si in the field. On the other hand, in the post-harvest life, fluctuations in the SS content of onion bulbs vary considerably between genetic materials and according to factors related to technology and pre- and post-harvest handling [4,5,22,23,52,53,54,55]. Ref. [4] suggested that the variations in SS content in onion bulbs during post-harvest storage were due to fluctuations in respiratory rates and endogenous content of soluble sugars.

The increase in pH by increasing salinity occurred due to increased intra- and intercellular concentrations of cations, especially Na^+^ [19]. In general, high cellular concentrations of cations trigger programs of synthesis for organic anions in the leaves for the buffering of excess cations absorbed by the roots [56]. In bulb formation, cations can be translocated from the leaves to the bulbs in ionic pairs with synthesized anions (especially malate), causing the pH reduction. On the other hand, the increase in bulb pH by the effect of shelf time is possibly due to the respiratory catabolism of organic acids using terminal oxidation to CO_2_ and H_2_O [51].

The increase in TA by increasing salinity occurred due to increased vacuolar concentrations of organic acids since only this analysis method identifies protonated forms of organic acids [56]. Ref. [57] reported that photorespiratory conditions and a high level of reducing pressure in the photosynthesis electron transfer system indicate the partial flow of the tricarboxylic acid (TCA) cycle, inducing the synthesis of organic acids and the consequent export of these acids from mitochondria to the vacuoles. Organic acids can supply electrons to the mitochondrial electron transport chain or can be accumulated for long periods as osmolytes or provide redox energy when needed [58]. Organic acids exhibit greater metabolic flexibility than large coenzymes, such as NADH and NADPH, and can transfer electrons and protons through membranes to other compartments.

In shelf life, the TA reduction in onion bulbs occurred due to the respiratory catabolism of organic acids to obtain the energy necessary for the processes related to the ripening and senescence of the bulbs using terminal oxidation of organic acids to CO_2_ and H_2_O, which also corroborates the increase in pH [51].

By increasing irrigation water salinity, the onion bulbs SS/TA ratio reduction occurred due to more soluble sugar catabolism than total titratable acids. In general, onion bulbs prefer to use soluble sugars as energy reserve forms to meet the higher ATP requirements, maintenance of redox balance, and enzymatic detoxification of lipid radical molecules and ROS caused by salt stress [12,30,38,39,45]. However, from the initial zero shelf-time (ST1) level to the subsequent levels ST2, ST3, and ST4, saline stress decreased rates of SS/AT ratio reduction, showing an increase in organic acid contribution to respiratory and redox metabolism of onion bulbs during their shelf life. The literature indicates the participation of organic acids in respiratory processes and mitochondrial redox regulation [51,57,58].

In the first shelf-life days, the salinity levels A2 and A3 led to a lower SS/TA ratio compared to the values observed at the control salinity level A1, indicating higher sugar consumption compared to titratable acids in these treatments. At the salinity level A4, we observed values lower than the control. The shelf time caused an increase in the SS/TA ratio of the onion, suggesting a preponderance in the titratable organic acid consumption compared to the sugars that make up the SS under high salinity. Therefore, these results show the organic acid consumption regulating the cellular redox of onion bulbs under salt stress.

The increase in the SS/TA ratio of the bulbs, as a function of Si supply, under salinity conditions, at ST1 possibly occurred because of improvements in an onion plants’ carbohydrate metabolism and osmotic adjustment. Previous studies have reported an increase in the soluble sugar concentration and osmotic regulation in plants treated with Si under salt and water stresses [46,49,50]. However, our results reveal that Si supply effect on the SS/TA ratio of onion bulbs occurred exclusively at ST1 (harvest). However, the absence of a significant effect of Si on the SS/TA ratio at ST2, ST3, and ST4 leads us to speculate on a possible transient accumulation mechanism of soluble sugars mediated by Si in onion bulbs for osmotic regulation of plants during the reproductive phenological period. Interestingly, [49] found that the increase in the soluble sugar concentration in cucumber plants occurred in the root system, accompanied by positive regulation of the expression of the aquaporin gene mediated by Si. However, further studies are needed to fully understand the effect of Si on the mechanism regulation related to sugar metabolism and osmotic control in onions pre- and post-harvest.

The increases in PyA in onion bulbs after harvest show significant changes in sulfur (S) metabolism in onion plants due to pre-harvest conditions of increases in salinity and Si fertilization. The modulation of activities and gene expression of rate-controlling enzymes in the metabolism of assimilation and absorption of S in plants by salinity stress have been previously reported [59]. We identified that Si supply alters the metabolism of S in different plant species, causing increases in S absorption and regulating the synthesis of amino acids and polyamines involved in stress response and tolerance [60,61]. Antioxidant defense related to the AsA-GSH cycle depends on the metabolic pathways of S [62]. Molecular domains containing thiol are, directly or indirectly, oxidized by ROS, generating relatively more stable oxidation products with modified physical conformations and biochemical activities. In addition, oxidized S-cysteine (S-Cys) chemical species, including sulfenic acid, glutathionylated Cys, sulfanilamide groups, and metal-sulfur bonds, are significant in redox signaling and regulation of ROS metabolism, causing direct effects on protein molecules, transcription factors and gene expression levels [12]. Interestingly, our results showed that salinity and Si supply significantly increased the concentration of sulfurous compounds in onion bulbs after harvest, up to a certain point, as shown by the increase in PyA concentration.

The increase of PyA in the first shelf life days and its subsequent reduction after 22.1 days may be due to the effects of concentration and degradation of thio-compounds that are precursors of pyruvic acid in onion bulbs, since we observed the following correlations: negative, between the PyA content and the mass loss of the bulbs; and positive, between the PCA content and bulb’s content of SS and TA.

AsA is a non-enzymatic antioxidant and redox buffer of many biological processes, including photosynthesis, ROS detoxification, and elimination of radical molecules [12,63,64,65,66,67,68,69]. There is strong evidence that adverse environmental conditions regulate AsA biosynthesis, including salinity stress [15,65,66]. AsA can directly eliminate the molecules of O_2_^•−^, ^•^OH, ^1^O_2_ or react with biologically generated radicals such as tocopheroxyl (that is, regeneration of tocopherols) and alkoxyl/peroxyl. In addition, AsA can reduce H_2_O_2_ molecules to H_2_O, acting as a cofactor in Mehler’s peroxidative reactions catalyzed by ascorbate peroxidase (APX). The AsA acts on the quenching of excess energy from the molecules of ^3^Chl* and ^1^O_2_ as a cofactor in the catalytic reaction of violaxanthin de-epoxidase (VDE), which converts violaxanthin into zeaxanthin [70,71,72,73,74]. The reason for the absence of a significant response of AsA to the increase in salinity at ST1 was possibly its dilution in bulbs still bloated in the harvest phase, not allowing the detection of significant variances by the adopted test. However, the means obtained at ST1 showed a slight increase in AsA (not significant) as a function of the increase in salinity. At the same time, the concentrations of AsA observed at ST2, ST3, and ST4 showed linear increases. Nevertheless, AsA content showed a positive correlation with the mass loss of the bulbs.

Storage time seems to concentrate AsA in onion bulbs treated with Si in pre-harvest without irrigation water salinity. The AsA concentration effect was possibly due to the increase in the mass loss of bulbs during storage and occurred exclusively in the control treatment (A1). This response shows that the AsA pool produced was sufficient to support the reactions involved in the natural processes of ripening and senescence. However, despite the increases in the AsA reservoirs in A2, A3, and A4 treatments under salt stress, in the first days of storage, AsA degradation occurred at the end of the onion’s shelf life. The increase in salinity reduced the shelf life of AsA, evidencing that salinity increased AsA consumption, possibly due to the increase of reactions related to the metabolism of cellular redox maintenance and catalysis of ROS and radical molecules generated biologically.

Studies conducted with *Acacia gerrardii* Benth and *Triticum aestivum*, under salinity stress and Si supply, showed significant improvements in AsA levels due to the supply of Si [75,76]. According to [76], the improvement in Si-mediated antioxidant defense occurs by modulation in enzymatic activity (including SOD, POD, CAT, APX, and GR) and biosynthesis of non-enzymatic antioxidants, including ascorbic acid, proline, and glycine betaine. We found that the increase in Si doses caused an increase in the AsA concentration of onion bulbs only at shelf times of 20, 40, and 60 days.

Although the AsA levels of onion bulbs, as a function of shelf time, reached higher peaks with the elevation of SD levels, we observed that the AsA peaks occurred at increasingly shorter shelf times as the SD levels increased. In many species, AsA regulates plant tolerance against factors of multiple abiotic stresses [65,77]. Ref. [65] reported that the redox state of ascorbate and the level of the apoplastic AsA pool affect the hormonal balance of plants, MAPK signaling cascades, and antioxidant enzymatic activities, which is therefore vital in the perception of environmental stress, redox homeostasis, and regulation of oxidative stress and plant physical-biochemical responses. However, the upstream mechanisms by which salinity and Si supply can regulate the activities and expression of the components of the antioxidant defense system in onions under storage conditions and the processes of senescence and ripening are not yet known. However, other studies, such as the description of proteomic and metabolomic profiles, gene identification, and PCR over time, are still needed to better understand the signaling mechanisms and responses to pre-harvest conditions of salt stress and Si supply in onion bulbs under storage conditions.

## 4. Materials and Methods

### 4.1. Location and Experimental Design

The experiment was conducted at the Rafael Fernandes Experimental Farm, belonging to the Federal Rural University of the Semi-Arid Region (UFERSA), located in the district of Alagoinha, Mossoró-RN, Brazil (5°03′37′′ S; 37°23′50′′ W and altitude of 72 m). The region recorded an average annual rainfall of 625 mm, with a dry season between June and January. The average annual air temperature has been recorded at a minimum of 21.3 °C and a maximum of 34.5 °C. The climate is classified as semi-arid (BSh), dry, and very hot, according to Köppen’s classification system [78]. The soil in the experimental area is an ARGISSOL, with a loamy-clay-sandy texture (726 g kg^−1^ of sand, 48 g kg^−1^ of silt, and 226 g kg^−1^ of clay, in the diagnostic B horizon). Table 1 shows a physicochemical analysis of the soil in the 0–20 cm layer.

**Figure 12:**
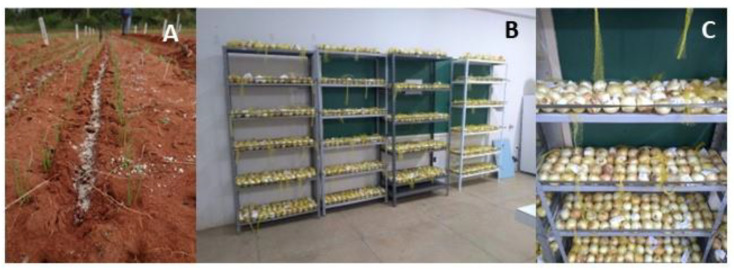
Si-fertilization (**A**) and onion storage (**B**,**C**).

The experimental design was in randomized blocks, arranged in a split-split plot scheme, with four replicates. The plots had four salinity levels of irrigation water (EC: 0.61; 1.74; 2.87, and 4.0 dS m^−1^), and the subplots had five levels of fertilization with silicon (SD: 0; 41.6; 83.2; 124.8, and 166.4 kg ha^−1^ of Si) and sub-sub plots lots had four conservation times (0, 20, 40, and 60 days after harvest), totaling 320 experimental units. The sample consisted of five onion bulbs per experimental unit, totaling 1600 bulbs [Classes 3 (50 ≤ Φ < 70 mm) and 4 (70 ≤ Φ < 90 mm)]. The samples were labeled and taken to the laboratory to evaluate the post-harvest quality of the bulbs within their respective conservation times. Figure 12 shows the details of Si-fertilization and onion storage.

### 4.2. Plant Material, Pre-Harvest Treatments, and Post-Harvest Storage

The experiment was carried out with onions (*Allium cepa* L.) sown on 16 July 2019. The genetic material used was the hybrid ‘Rio das Antas’, a common cultivar in the northeastern semi-arid region of Brazil. Cultivation, fertilization, and irrigation were performed according to local recommendations, crop needs, and availability of water and nutrients in the soil [79,80,81,82].

The treatments were of four salinity levels of irrigation water (0.61; 1.74, 2.87, and 4.0 dS m^−1^) and five levels of fertilization with silicon (0; 41.6; 83.2, 124.8, and 166.4 kg ha^−1^ of Si), arranged in plots and subplots, respectively. Water salinity was elevated by the addition of sodium chloride, calcium chloride, and magnesium sulfate salts in a molar charge ratio of 7:2:1. Fertilization with Si was carried out at planting, with a fertilizer based on natural diatomaceous earth derived from the species Melosira granulata—Agrisilica^TM^ with 2 mm diameter (26% Si; 0.07% N; 0.02% P; 0.08% K; 0.09% S; 1.4% Ca; 1.1% Mg; 1.3% Fe; 219 mg kg^−1^ Mn; < 5 mg kg^−1^ B; 22 mg kg^−1^ Cu; 18 mg kg^−1^ Zn; and 2.1 mg kg^−1^ Mo), produced by Agripower Australia Limited.

Harvest was carried out on 16 December 2019, after a natural curing process of the bulbs, for 10 days, under field conditions. The beginning of the curing process was defined when 70% of the plant population was physiologically mature (leaves fallen over) when the suspension of the water supply by irrigation was also determined. The bulbs harvested were selected according to the class of commercial diameter, between 50 and 70 mm, and then placed on shelves, in polyethylene nets (10 mm mesh), for 0, 20, 40, and 60 days, under ambient storage conditions, at an average air temperature of 29.5 ± 0.7 °C and relative humidity of 67 ± 5%, were obtained by digital thermo-hygrometer (Jprolab^®^, São José dos Pinhais, Brazil).

### 4.3. Evaluated Characteristics

The morphological analyses of the plants were bulb firmness (BF), mass loss (ML), and color (CLR) of onion bulbs, determined at 0, 20, 40, and 60 days after harvest (DAH). BF and CLR analyses were performed separately in each of the bulbs in the sample, recording the mean of the five bulbs for each treatment replicate. ML was determined by percentage ratio, recording the initial and final weights of each sample set of five bulbs.

The post-harvest quality of the bulbs was evaluated by determining the levels of soluble sugar (SSg), total soluble solids (SS), total titratable acidity (TA), and SS/TA ratio, as well as pH and the concentrations of pyruvic (PyA) and ascorbic (AsA) acids. The evaluations were performed in homogenized juice from the five onion bulbs of the sample, obtained using a centrifugal juice extractor 700 W with stainless steel blades (Philips Walita^®^, Brazil).

#### 4.3.1. Evaluation of Bulb Firmness and Mass Loss

Bulb firmness, measured in Newton force (N), was evaluated using a penetrometer (Lutron^®^ PTR-300, Taiwan), with a tip of 8 mm in diameter and penetration at a depth of 7 mm. The readings were performed in the middle equatorial portion of the bulbs at two equidistant points, on opposite sides, after removing the dry tunic from the bulbs.

Mass loss was obtained using the percentage ratio between the weight of onion bulbs at the initial shelf time (0 DAH) and the weight of the bulbs at their respective shelf times, at the moment of evaluation, according to the following equation:

(1) $$\mathrm{ML}\;\left(\%\right)=\left(\frac{M_{i}}{M_{f}}-1\right)\times 100\times \left(-1\right)$$

where;

ML is the mass loss, in %;

M_i_ is the mass of fresh matter of the sample at the beginning of storage, in g; and

M_f_ is the mass of fresh matter of the sample at the evaluated shelf time, in g.

#### 4.3.2. Evaluation of Bulb Color

Bulb color was evaluated from the color sensation recommended by the International Lighting Commission (*Commission Internationale de l’Eclairage*—CIE), quantified by the determination of color parameters L* (lightness or brightness), a* (color variation between green and red), b* (color variation between blue and yellow), C* (chromaticity or saturation) and °H (hue or hue angle). The CIE coordinates L*, a*, and b* were obtained by a colorimeter (Konica Minolta^®^ CR-410, Japan) (variation of Y reflectance: from 0.01% to 160.00%), with a silicon photocell detector and light source in the form of a xenon flashtube, using a wide-area illumination color measuring head, reflecting directly in the upper polar region of the onion bulbs, covering a circular area of 50 mm in diameter. The standard observer corresponds to Standard 2° CIE 1931 ($\bar{x}$2λ, ȳλ, ȳλ).

The CIE color spaces C* and H* were calculated from the CIE coordinates L*, a*, and b* using the following mathematical applications [83]:

(2) $$C^{*}=\sqrt{a^{*}{}^{2}+{\;b}^{*}{}^{2}}$$

(3) $$h^{*}=180+\left[\frac{\left(\arctan\frac{b^{*}}{a^{*}}\right)}{6.2832}\right]\times 360,{\;\mathrm{when}\;a}^{*}<0$$

(4) $$h^{*}=\left[\frac{\left(\arctan\frac{b^{*}}{a^{*}}\right)}{6.2832}\right]\times 360,{\;\mathrm{when}\;a}^{*}>0$$

#### 4.3.3. Evaluation of Soluble Sugars (SSg), Soluble Solids (SS), Titratable Acidity (TA), SS/TA Ratio, and Hydrogen Potential (pH)

The soluble sugar (SSg) and titratable acidity (TA) content were evaluated in onion bulbs according to the methods described by [84,85], respectively. SSg was extracted from 1 g samples of juice from fresh bulbs and dissolved in 100 mL of pure demineralized water. The determinations were carried out by colorimetry from 50 μL aliquots taken from the extracts. For the readings, the extract aliquots (50 μL) were mixed with 950 μL of pure water and 2000 μL of anthrone reagent (2 g L^−1^ of H_2_SO^4^), heated (water bath at 100 °C for 8 min), and cooled (ice bath). The readings were recorded in the spectral range of 620 nm, and SSg content was calculated from a standard glucose curve. TA was determined from 1 g samples of onion bulb juice dissolved in 50 mL of pure demineralized water by titration with 0.1 N NaOH and phenolphthalein indicator at 1%.

The soluble solids (SS) content and the hydrogen potential (pH) of onion bulbs were determined directly in the juice using a portable digital Brix refractometer (Dbr-92, China), converted to the Brix scale (%), and an electrical potentiometer (mV), converted to the pH scale. The device used to measure SS was the DBR45 digital refractometer (refractive index of 1.3330–1.4098), with automatic temperature compensation, while the device used to measure pH was the benchtop pH meter (Hanna^®^ Instruments HI 2221, United States), with HI-1131B pH electrode and HI 7662 automatic temperature compensation probe.

SS/TA ratio was obtained through the mathematical ratio between the observed values of SS and TA.

#### 4.3.4. Evaluation of Pyruvic Acid and Ascorbic Acid Concentrations

Pyruvic acid (PyA) and ascorbic acid (AsA) concentrations were evaluated in onion bulbs according to the methods described by [86,87], respectively. PyA was extracted from 0.5 g samples of juice from fresh bulbs, dissolved in 1.5 mL of 5% trichloroacetic acid (TCA) and 18 mL of pure demineralized water. The determinations were carried out by colorimetry, using 1 mL aliquots taken from the extracts. For readings, the extract aliquots (1 mL) were mixed with 1 mL of 2,4-dinitrophenylhydrazine (2.4-DNPH), 0.125 g L^−1^ of 2 N HCl, and 1 mL of pure water and then heated (water bath at 37 °C for 10 min), cooled (ice bath) and mixed with 5 mL of 0.6 N NaOH. Readings were recorded in the spectral range of 420 nm, and the PyA content was calculated from a standard sodium pyruvate curve. AsA was extracted from 1 g samples of onion bulb juice, dissolved in 50 mL of 0.5% oxalic acid. The determinations were carried out by titration with Tillman solution (2,6-dichlorophenol indophenol) at 0.02% (refrigerated) from 5 mL of the extract dissolved in a volume of 50 mL based on pure demineralized water. The content was calculated by proportional ratios based on the titration of a standard ascorbic acid solution.

### 4.4. Statistical Analysis

The results obtained were subjected to analysis of variance and polynomial regression tests, and then polynomial regression equations were fitted using linear and nonlinear regression models. The criteria for selecting the models were biological meaning, the significance of the estimators of regression parameters, and R^2^ values.

## 5. Conclusions

The present research results show that the increase in salinity of irrigation water and shelf time reduces the quality of onion bulbs, causing reduced firmness and significant mass loss. At the same time, fertilization with Si did not contribute significantly to improving these variables. On the other hand, salinity and Si do not considerably affect the tunic color of onion bulbs, showing that onions grown under such environmental conditions have appeared for sale. In addition, our studies have shown that increased salinity causes a reduction in the content of sugars and total soluble solids of onion bulbs, as well as pH and SS/TA ratios, increasing the concentrations of titratable acids and pyruvic acid along with shelf life. Onion bulbs grown under salinity conditions may exhibit a more astringent and acidic flavor to the consumer, especially in prolonged permanence under shelf conditions.

In contrast, the increase in Si fertilization with doses between 121.8 and 127.0 kg ha^−1^ contributes to increasing the content of sugars and total soluble solids and reducing the SS/TA ratio, especially at 20 shelf-life days, with only a slight increase in pyruvic acid concentration. Si fertilization can improve the flavor qualities of onions grown under salinity, promoting a better balance between the astringency and sweetness of bulbs after cooking or improving the aromatic background of salads and culinary dishes by increasing pungency expression. Although salinity results in losses of palatable quality in onion bulbs, it can promote the increased concentration of ascorbic acid during shelf life until approximately 36 days of storage. Additionally, the increase in fertilization with Si, up to the maximum dose of 166.4 kg ha^−1^, also promotes an increased concentration of ascorbic acid in bulbs during shelf life until approximately 40 days of storage. Thus, the increase in irrigation water salinity and the management of Si fertilization promotes the biofortification of onions with vitamin C. Therefore, our results can help define guidelines for fertilization with Si in the cultivation of onions under salinity conditions, focusing on developing new markets based on the quality of products and the quality of life for consumers.

## Figures and Tables

**Table 1:** Physicochemical soil of the experimental area in the 0–20 cm layer.

pH	Ca^2+^	Mg^2+^	K^+^	Na^+^	Al^3+^	H^+^+Al^3+^	P	M.O.	Si	Clay	Silt	Sand
	**───────────cmol_c_dm^−3^───────────**						**mg dm^−3^**	**g kg^−1^**	**mg dm^−3^**	**--------g kg^−1^--------**		
7.30	1.60	0.41	0.14	0.07	0.00	0.44	31.08	4.65	0.10	29	21	950

pH in water (1:2.5); Ca^2+^, Mg^2+^, and Al^3+^: 1 mol L^−1^ KCl extractor; K^+^ and P: Mehlich^−1^ extractor; H^+^+Al^3+^: SMP extractor; M.O.: organic matter by Walkey–Black; Si: 0.05 mol L^−1^ acetic acid extractor.

## Data Availability

All data are presented in the paper.
